# Does time to esophagectomy following neoadjuvant immunochemotherapy for locally advanced esophageal squamous cell carcinoma affect outcomes?

**DOI:** 10.3389/fimmu.2022.1036396

**Published:** 2022-10-14

**Authors:** Zhi-Nuan Hong, Zhixin Huang, Kai Weng, Jihong Lin, Mingqiang Kang

**Affiliations:** ^1^Department of Thoracic Surgery, Fujian Medical University Union Hospital, Fuzhou, China; ^2^Key Laboratory of Cardio-Thoracic Surgery (Fujian Medical University), Fujian Province University, Fuzhou, China; ^3^Key Laboratory of Ministry of Education for Gastrointestinal Cancer, Fujian Medical University, Fuzhou, China; ^4^Fujian Key Laboratory of Tumor Microbiology, Fujian Medical University, Fuzhou, China

**Keywords:** neoadjuvant immunochemotherapy, esophageal squamous cell carcinoma, time to surgery, minimally invasive esophagectomy, disease free survival

## Abstract

**Objectives:**

Neoadjuvant immunochemotherapy (nICT) is a novel pattern for locally advanced esophageal squamous cell carcinoma (ESCC), and the time to surgery (TTS) is recommended as 4-6 weeks. However, there were some patients with prolonged TTS(> 6 weeks). This study aimed to explore whether prolonged TTS (> 6 weeks) would affect the outcomes.

**Methods:**

Patients diagnosed with locally advanced ESCC between January 2020 and March 2022 and undergoing esophagectomy following nICT were identified based on a prospectively collected database. Primary outcome measures were pathological complete response (pCR) and disease-free survival (DFS), and the secondary outcomes were 30-day postoperative mortality and morbidity, surgical time, postoperative hospital stay, and hospital expense.

**Results:**

Total of 95 patients were included for analysis, with 52 patients in the standard TTS group and 43 patients in the prolonged TTS group. The clinical and demographic characteristics of the two groups were comparable. The prolonged group had a median 18 days longer TTS(P<0.001). The pCR rate was 23.08% (12/52) in the standard group and 16.28% (7/43) in the prolonged group (P=0.41). Multivariate regression analysis further indicated that TTS wasn’t an independent factor in predicting pCR (P=0.41). The median follow-up time was 10.5 months in the standard TTS group and 11.2 months in the prolonged TTS group. A total of five recurrences occurred with two events in the standard TTS group and three events in the prolonged TTS group, and no significant difference was observed in DFS(P=0.60). Both groups were comparable in postoperative hospital stays, total hospital stay, hospital expenses, and comprehensive complications index (CCI). The complications and major complications were also similar in both groups. Spearman test further indicated that there was no linear correlation among TTS with hospital expenses, postoperative hospital stays, hospital stay, CCI index, lymph nodes moved number, or surgical time, with a p-value of 0.48, 0.63, 0.80, 0.92, 0.09, 0.38 respectively.

**Conclusions:**

Based on present evidence, TTS after completion of nICT is not of major importance concerning pathological response, disease-free survival, and short-term postoperative outcomes.

## Background

The latest data from The National Cancer Center of China show that esophageal cancer ranks sixth in incidence and fourth in mortality. In China, more than 90% of patients have esophageal squamous cell carcinoma (ESCC) (1, 2). Currently, the standard treatment for locally advanced ESCC is neoadjuvant chemoradiotherapy following minimally invasive esophagectomy (3). Neoadjuvant chemotherapy is prevalent in China and Japan (4, 5). Neoadjuvant chemotherapy (nCT) or neoadjuvant chemoradiotherapy (nCRT) plus esophagectomy could both improve long-term survival. However, the treatment of ESCC is still challenging due to the high recurrence and unpromising long-term survival.

Results from the CheckMate577 study showed that postoperative adjuvant immunotherapy extended the median disease-free survival of patients with esophageal cancer after nCRT following esophagectomy (11.0 months) (95%CI: 8.3-14.3 months) (6). Further, several clinical trials have indicated that immunotherapy plus nCT in locally advance ESCC has a favorable pathological complete response(pCR) rate with acceptable toxicity (7, 8). Neoadjuvant immunochemotherapy(nICT) is a novel and promising pattern to treat locally advanced ESCC. The optimal interval from the completion of the last cycle of nICT to esophagectomy (defined as time to surgery, TTS) is one issue to be clarified. At present, the interval is recommended as 4-6 weeks, referring to the treatment mode of neoadjuvant chemoradiotherapy and neoadjuvant chemotherapy. However, there are a significant proportion of patients, either for administrative reasons or because of treatment-related adverse events or the influence of COVID-19, accept esophagectomy at a longer interval (>6 weeks). There is, to date, still no study addressing the effect of TTS after nICT, on pCR rate, disease-free survival (DFS), and postoperative outcomes after esophagectomy. The hypothesis of this study was that prolonged TTS (>6 weeks) would increase the pCR rate and improve the DFS without increasing the risk of postoperative complications compared with standard TTS (4-6 weeks).

## Methods

### Patient selection and study design

This is a retrospective study based on prospectively collected data. Consecutive patients who underwent esophagectomy followed nICT for ESCC at our department from January 2020, and March 2022 were identified. The inclusion criteria included: diagnosed with ESCC; undergoing 2-4 cycles of nICT following radical esophagectomy; clinical staged with cT1N1-3M0 or cT2-4aN0-3M0(II-IVA); with ASA status≦III. The exclusion criteria included: patients with nonresectable tumors or metastases during exploratory surgery; patients receiving neoadjuvant chemoradiotherapy or neoadjuvant chemotherapy alone or in combination with other targeted therapy; patients with palliative surgery; patients with jejunostomy alone. We have finished two phaseII clinical trials, and most patients in this study were included in clinical trials. Only a few patients weren’t included in clinical trials who received neoadjuvant immune-combined chemotherapy in other hospitals and then underwent surgery in our department. This study was approved by the Ethics Committee from Fujian Medical University Union Hospital.

### Neoadjuvant treatment protocols

Patients in the NICT group received at least one cycle (mostly two cycles) of intravenous PD-1 inhibitors (sintilimab dose 200 mg, pembrolizumab dose 200 mg, toripalimab dose 240 mg, tirelizumab dose 240mg, and camrelizumab dose 200 mg) every three weeks (1 day) in a combination of platinum chemotherapy drugs and paclitaxel/docetaxel. The details of neoadjuvant treatment protocols were listed in our previous reports (9, 10). When immunotherapy-related adverse events above grade 2 occur, we stop applying nICT and give priority to dealing with adverse events. For patients who finished 2 or 3 cycles of nICT, we conducted another clinical evaluation followed by multidisciplinary consultation to determine whether the patient should undergo esophagectomy or continue treatment.

### Surgery protocols

For patients suitable for radical esophagectomy after clinical evaluation, the interval to surgery was recommended as 4–6 weeks. The interval was defined as from the completion of the last cycles of neoadjuvant therapy to the operation day. However, there were still patients with prolonged TTS(> 6 weeks) due to either administrative reasons or treatment-related adverse events, or the influence of COVID-19.

All patients received McKeown minimally invasive esophagectomy (MIE) with thoracoscopic-assisted or with robot-assisted. We regularly conducted 2-field lymphadenectomy. When there were swollen lymph nodes in the neck in preoperative clinical evaluation, standard three-field lymphadenectomy was performed. The gastric tube was 3.0-3.5cm, and left cervical anastomosis was performed using a circular stapler. Jejunostomy was regularly conducted during the abdominal procedure.

### Outcome measures

The primary outcomes were the pathological response. The pathological TNM stage was staged according to the 8th edition American Joint Committee on Cancer/Union for International Cancer Control staging system. The pathological complete response (pCR) was defined as no residual tumor cells in both tumor and lymph nodes. Disease-free survival (DFS) was defined as the time from surgical resection to local recurrence. The secondary outcomes included 30-day postoperative mortality and morbidity, operation time, postoperative hospital stay, and hospital expense. We used the Clavien-Dindo classification to grade complications, and the Clavien-Dindo classification grade≧ 3a was considered as major complications. To systematically assess the complications, we also applied the comprehensive complications index **(**CCI), which was developed based on the Clavien-Dindo classification system, and was calculated on an online website (www.assessurgery.com) (11).

### Statistical analysis

We divided patients into two groups: the standard group (with TTS 4-6 weeks) and the prolonged group (with TTS > 6 weeks). The continuous variables of the abnormal distribution were represented by the median (quartile range), and the continuous variables of the normal distribution were represented by the mean (standard deviation, SD). The categorical variables were expressed as numbers (percentages).

For equivalent variables with a normal distribution, an independent Student’s test was used. The Mann-Whitney U test was used to compare the abnormal distribution variables between the two groups. The frequency of the classification variables was determined by using the Chi-squared test or Fisher’s exact test, where appropriate. Univariate and multivariate regression analyses were conducted to further determine whether the TTS could improve the pCR rate. ROC (receiver operator characteristic curve) was drawn to evaluate the predictive ability of TTS for pCR. The correlation between TTS with clinical outcomes was determined by the Spearman test or Pearson test, where appropriate. Statistical analysis was performed in R Version 4.0.4(R Foundation for Statistical Computing, Vienna, Austria). P<0.05 was considered a significant difference.

## Results

### Patient selection and baseline characteristics

**Figure 1:**
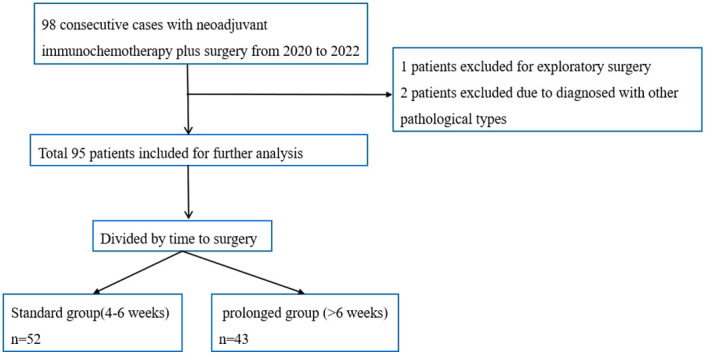
Flowchart of patient selection.

The patient selection details were showed in **Figure 1**. Total of 95 patients was included for further analysis, with 52 patients in the standard group and 43 patients in the prolonged group. The clinical and demographic characteristics of the two groups were well balanced, including age, sex, body mass index (BMI), diabetes, drinking history, smoking history, American Society of Anesthesiologists (ASA) status, tumor location, forced expiratory volume in one second (FEV1), ejection fractions (EF), lymphadenectomy filed, clinical stage, route of gastric conduit, procedure type, clinical stage, neoadjuvant cycles, and types of neoadjuvant chemotherapy. The prolonged group had a median 18 days longer TTS(P<0.001), with 52(45,63) days in the prolonged group and 34 (30,38) days in the standard group, respectively. The comparison of baseline characteristics between the standard group and the prolonged group is summarized in **Table 1**.

**Table 1:** The comparison of baseline characteristics between the standard group and the prolonged group is summarized in Table 1.

Contents	total (n=95)	prolonged group (n=43)	standard group (n=52)	p
age, mean (SD)	60.45(6.72)	60.86(5.89)	60.12(7.32)	0.59
sex, n (%)				0.44
female	23(24.21)	12(27.91)	11(21.15)	
male	72(75.79)	31(72.09)	41(78.85)	
body mass index, median [IQR]	21.48[20.37,22.77]	21.48[19.92,22.86]	21.67[20.39,22.67]	0.75
diabetes, n (%)	6(6.32)	2(4.65)	4(7.70)	0.54
drinking history, n (%)	32(33.68)	12(27.91)	20(38.46)	0.28
Smoking history, n (%)	54(56.84)	24(55.83)	30(47.69)	0.85
EF%, mean (SD)	67.17(5.52)	67.01(6.10)	67.313(4.99)	0.79
FEV1, mean (SD)	2.58(0.64)	2.47(0.60)	2.671(0.66)	0.14
clinical stage, n (%)				0.71
II	40(42.11)	19(44.19)	21(40.39)	
III-IVA	55(57.90)	24(55.81)	31(59.62)	
ASA status, n (%)				0.89
II	88(92.63)	40(93.02)	48(92.31)	
III	7(7.37)	3(6.98)	4(7.69)	
tumor location, n (%)				0.36
upper	9(9.474)	3(6.98)	6(11.54)	
middle	49(51.58)	20(46.51)	29(55.77)	
lower	37(38.95)	20(46.51)	17(32.69)	
neoadjuvant chemotherapy				
PF	85(89.47)	39(90.70)	46(88.46)	0.72
DF	10(10.53)	4(9.30)	6(11.54)	
neoadjuvant treatment cycles, n (%)				0.25
<3	65(68.42)	32(74.42)	33(63.46)	
≥3	30(31.58)	11(25.58)	19(36.54)	
PD-1 inhibitors				0.78
pembrolizumab	23(24.21)	11(25.58)	12(23.08)	
others	72(57.79)	31(74.42)	40(76.92)	
thoracic procedure				0.49
Video-assisted	79(83.16)	37(86.05)	42(80.77)	
Robotic- assisted	16(16.84)	6(13.95)	10(19.23)	
way-up procedure, n (%)				0.23
posterior mediastinal	87(91.58)	41(95.35)	46(88.46)	
restro-sternal	8(8.42)	2(4.65)	6(11.54)	
lymphadenectomy, n (%)				0.26
2-field	82(86.32)	39(90.70)	43(82.69)	
3-field	13(13.68)	4(9.30)	9(17.31)	
Time to surgery, median [IQR]	42[34,50]	52[45,63]	34[30,38]	<0.001

ASA, American Society of Anesthesiologists; FEV1, forced expiratory volume in one second; EF, ejection fractions.

### Comparisons of short-term outcomes

All patients in the standard group and the prolonged group successfully underwent minimally invasive esophagectomy (MIE) without conversion into thoracotomy. Compared with the standard group (median 326min), there was a reduction in surgical time in the prolonged group (median 315min), but without a significant difference. The prolonged group also had a slight reduction in hospital expenses, postoperative hospital stays, and thoracic tube stay(P>0.05). The details of short-term outcomes are summarized in **Table 2**.

**Table 2:** Comparisons of perioperative outcomes between prolonged group and standard group.

Contents	total (n=95)	prolonged group (n=43)	standard group (n=52)	p
surgical time (min), median [IQR]	317 [283,373]	315 [285,374]	326 [280,361]	0.73
Intraoperative blood loss (ml), median [IQR]	100 [80,100]	100 [100,100]	100 [50,150]	0.84
expenses, median [IQR]	8.68 [7.87,10.52]	8.65 [7.74,10.52]	8.88 [8.02,10.57]	0.52
postoperative hospital stays (days), median [IQR]	11 [8,13]	10 [8,13]	11 [8,13]	0.44
hospital stays, median [IQR]	16 [13,25]	17 [13,27]	16 [13,24]	0.97
thoracic tube stays, mean (SD)	11.39 (8.51)	10.44 (5.64)	12.20 (10.26)	0.33
LN moved number, median [IQR]	36 [29,42]	34 [28,40]	38 [30,42]	0.43
CCI index, mean (SD)	25.74 (14.59)	25.43 (14.48)	25.99 (14.67)	0.85
ypT0N0, n (%)				
No	76 (80.00)	36 (83.72)	40 (76.92)	0.41
Yes	19 (20.00)	7 (16.28)	12 (23.08)	
ypT0N+, n (%)				
No	67 (70.53)	31 (72.09)	36 (69.23)	0.76
Yes	28 (29.47)	12 (27.91)	16 (30.77)	
ypstage, n (%)				0.72
I	39 (41.05)	18 (41.86)	21 (40.39)	
II	16 (16.84)	8 (18.61)	8 (15.39)	
IIIa	17 (17.90)	9 (20.93)	8 (15.39)	
IIIb	22 (23.16)	8 (18.61)	14 (26.92)	
IVa	1 (1.05)	0 (0.00)	1 (1.92)	
TRG grade				0.82
0	28 (29.47)	12 (27.91)	16 (30.77)	
1	16 (16.84)	8 (18.61)	8 (15.39)	
2	21 (22.11)	8 (18.61)	13 (25.00)	
3	30 (31.58)	15 (34.88)	15 (28.85)	

CCI, comprehensive complications index; LN, lymph nodes.

The CCI was comparable in both groups, with 25.43(14.48) in the prolonged group and 25.99(14.67) in the standard group. The incidence of pneumonia, pleural effusion, anastomotic leakage, chylothorax, and postoperative blood transfusion were also comparable in both groups. Major complications were comparable in both groups.

Further, 30-day mortality, 30-day readmission rate, and ICU readmission rate were also similar in both groups. The details of postoperative complications are summarized in **Table 3**.

**Table 3:** Comparisons of postoperative complications within 30-day after operation evaluated by Clavien-Dindo classification between prolonged group and standard group.

contents	total (n=95)	prolonged group (n=43)	standard group (n=52)	p
electrolyte disorder, n (%)				0.75
0	58 (61.05)	27 (62.79)	31 (59.62)	
1	37 (38.95)	16 (37.21)	21 (40.39)	
analgesic, n (%)				
0	68 (71.58)	31 (72.09)	37 (71.15)	0.92
1	27 (28.42)	12 (27.91)	15 (28.85)	
vomiting, n (%)				0.67
0	92 (96.84)	42 (97.67)	50 (96.15)	
1	3 (3.16)	1 (2.33)	2 (3.85)	
pleural effusion, n (%)				
≥2	18 (18.95)	8 (18.61)	10 (19.23)	0.94
≥3	11 (11.58)	6 (13.95)	5 (9.62)	0.51
bleeding, n (%)				
≥1	32 (33.68)	17 (39.54)	15 (28.85)	0.27
≥2	2 (2.11)	0 (0.00)	2 (3.85)	0.19
≥3	1 (1.05)	0 (0.00)	1 (1.92)	0.37
chylothorax, n (%)				0.67
0	92 (96.84)	42 (97.67)	50 (96.15)	
1	3 (3.16)	1 (2.33)	2 (3.85)	
cardiac events, n (%)				
≥1	19 (20.00)	5 (11.63)	14 (26.92)	0.06
≥2	15 (15.79)	4 (9.30)	11 (21.15)	0.12
anastomic leakage, n (%)				
≥2	9 (9.47)	3 (6.98)	6 (11.54)	0.45
≥3	1 (1.05)	1 (2.33)	0 (0.00)	0.27
pneumonia, n (%)				
≥2	56 (58.95)	23 (53.49)	33 (63.46)	0.33
≥3	40 (42.11)	20 (46.51)	20 (38.46)	0.43
30-day mortality, n (%)				1.00
no	95 (100.00)	43 (100.00)	52 (100.00)	
yes	0 (0.00)	0 (0.00)	0 (0.00)	
30-day readmission, n (%)				0.41
no	91 (95.79)	42 (97.67)	49 (94.23)	
yes	4 (4.21)	1 (2.326)	3 (5.77)	
ICU readmission, n (%)				0.11
no	92 (96.84)	43 (100.00)	49 (94.23)	
yes	3 (3.16)	0 (0.00)	3 (5.77)	

**Figure 2:**
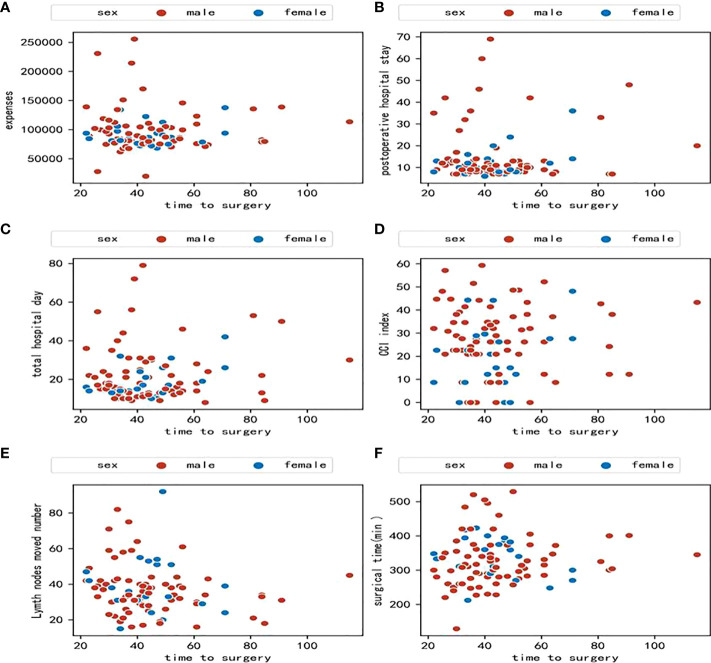
**(A)** Scatter diagram between time to surgery and hospital expenses; **(B)** Scatter diagram between time to surgery and postoperative hospital stay; **(C)** Scatter diagram between time to surgery and total hospital stay; **(D)** Scatter diagram between time to surgery and comprehensive complications index **(**CCI); **(E)** Scatter diagram between time to surgery and lymph nodes moved number; **(F)** Scatter diagram between time to surgery and surgical time.

**Figure 3:**
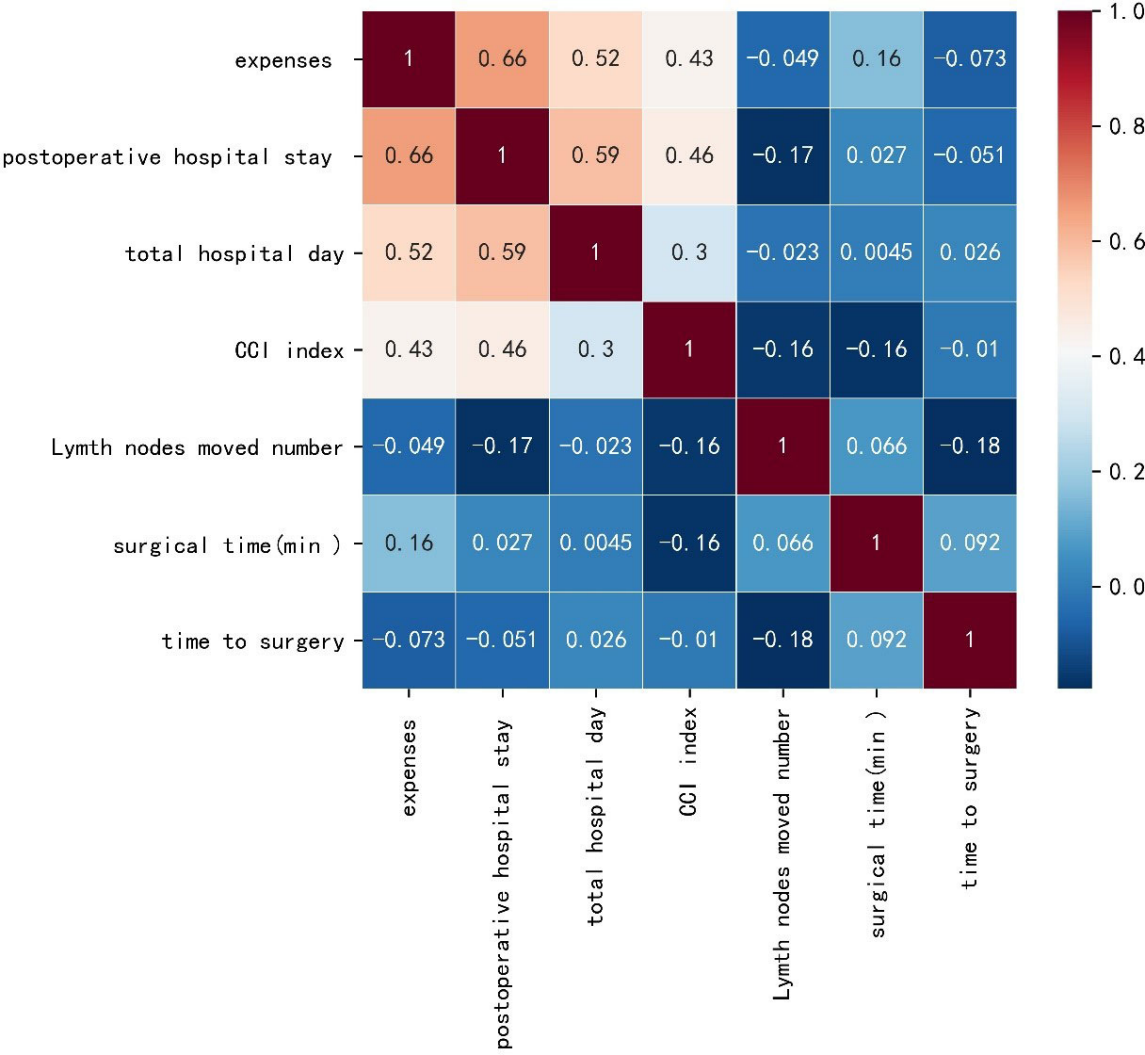
Heat map based on the Spearman test of time to surgery with hospital expenses, postoperative hospital stays, hospital stay, comprehensive complications index (CCI) index, lymph nodes moved number and surgical time.

A preliminary scatter diagram indicated that there was no linear correlation among TTS with hospital expenses, postoperative hospital stays, hospital stay, CCI index, lymph nodes moved number, or surgical time (**Figure 2**). Spearman test also indicated that there was no linear correlation among TTS with hospital expenses, postoperative hospital stays, hospital stay, CCI index, lymph nodes moved number, or surgical time, with a p-value 0.48, 0.63, 0.80, 0.92, 0.09, 0.38 respectively. The absolute value of the correlation coefficient among TTS with hospital expenses, postoperative hospital stays, hospital stay, CCI index, lymph nodes moved number, or surgical time was all below 0.10, which indicated a very weak correlation (**Figure 3**).

### Comparisons of pathological response and disease-free survival

The pCR rate in both tumor and lymph nodes was 23.08% (12/52) in the standard group and 16.28% (7/43) in the prolonged group (P=0.41). The pCR rate in tumors only was 30.77% (16/52) in the standard group and 27.91% (12/43) in the prolonged group(P=076). There was no significant distribution of ypTNM stage and TRG grade between the standard group and prolonged group either, with P values 0.72 and 0.82, respectively. The AUC (areas under the ROC curve) of TTS for ypT0N0 and ypT0N+ was 0.47 and 0.45, respectively, which indicated weak predictive power. Multivariate regression analyses showed that TTS was not an independent factor in predicting pCR(P=0.41). Only the clinical stage was identified as an independent factor in predicting pCR. Compared with patients in clinical stage II, a patient diagnosed with clinical stage III-IVA had less possibility of achieving pCR, with an odds ratio value of 0.32 (P=0.04). The details of univariate regression analyses and multivariate regression analyses are summarized in **Table 4**.

**Table 4:** The details of univariate regression analyses and multivariate regression analyses for ypT0N0.

Contents	univariate analysis			multivariate analysis		
	OR	95%CI	p	OR	95%CI	p
body mass index	0.92	(0.70, 1.16)	0.50	0.94	(0.75,1.17)	0.57
clinicalstage II	Reference			Reference		
clinical stage III-IVA	0.32	(0.10, 0.95)	0.04	0.34	(0.12,0.97)	0.04
tumor location Upper	Reference			Reference		
tumor location Middle	0.99	(0.16, 8.38)	0.99	1.01	(0.18,5.60)	0.99
tumor location Lower	1.00	(0.15, 9.24)	1.00	0.68	(0.11,4.09)	0.67
without diabetes	Reference			Reference		
with diabetes	1.58	(0.06, 18.28)	0.74	0.79	(0.09,7.18)	0.83
without drinking history	Reference			Reference		
with dringking history	0.75	(0.20, 2.71)	0.66	0.89	(0.30,2.61)	0.83
without smoking history	Reference			Reference		
with smoking history	1.48	(0.37, 6.97)	0.59	1.06	(0.38,2.92)	0.92
ASA status II	Reference			Reference		
ASA status III	0.74	(0.03, 7.49)	0.81	0.65	(0.07,5.73)	0.70
age<65 years old	Reference			Reference		
age≥65 years old	0.65	(0.17, 2.23)	0.51	0.77	(0.25,2.40)	0.66
Female	Reference			Reference		
Male	0.76	(0.14, 3.99)	0.74	0.87	(0.28,2.74)	0.81
Time to surgery(4-6 weeks)	Reference			Reference		
Time to surgery>6 weeks	0.57	(0.18, 1.73)	0.33	0.65	(0.23,1.83)	0.41
PF regimens	Reference			Reference		
DF regimens	0.29	(0.01, 2.13)	0.29	0.41	(0.05,3.48)	0.42
neoadjuvnat treatment cycles<3	Reference			reference		
neoadjuvnat treatment cycles≥3	1.49	(0.43, 5.08)	0.53	1.34	(0.47,3.85)	0.58

**Figure 4:**
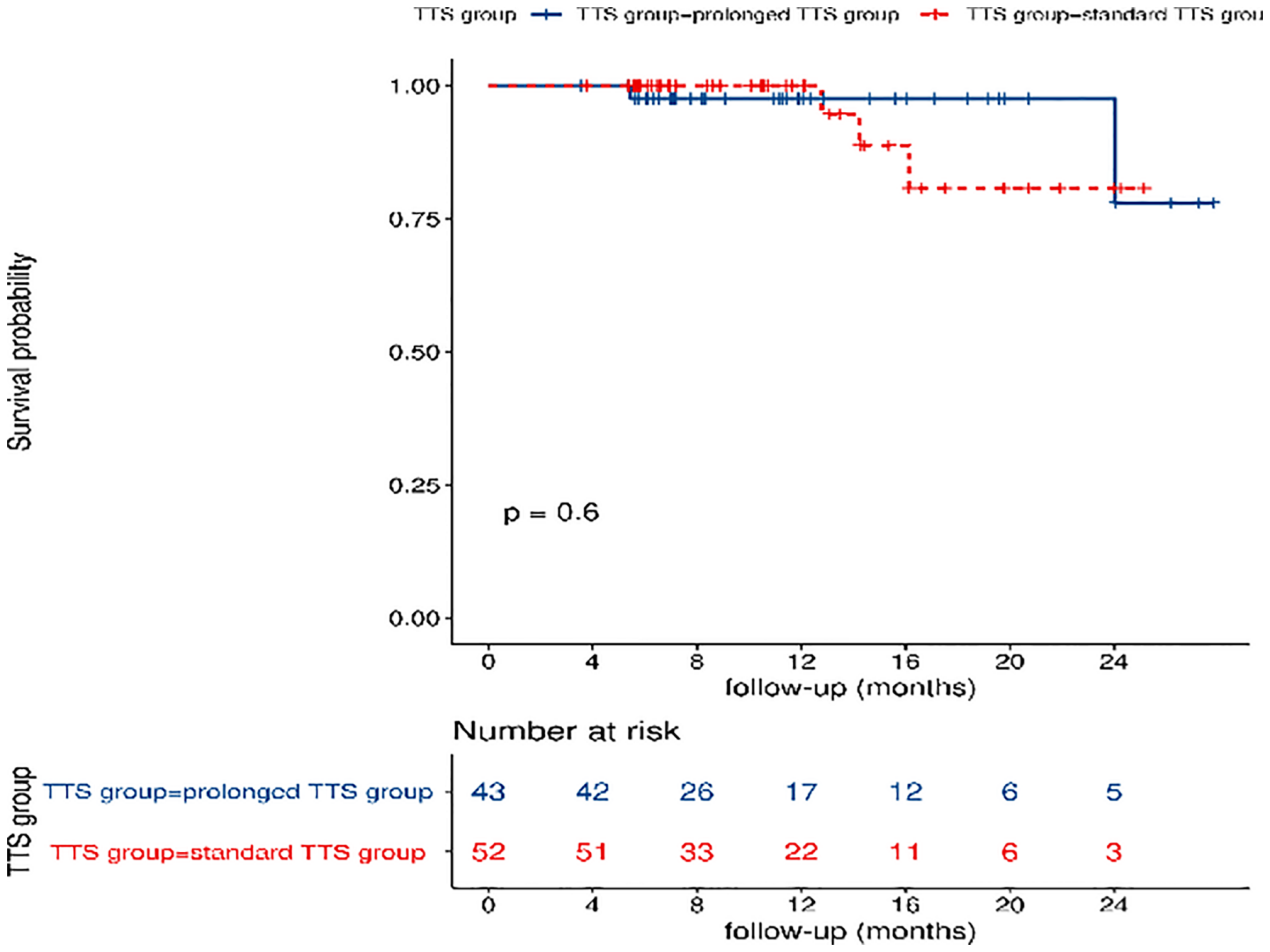
Comparisons of disease-free survival between the standard TTS group and the prolonged TTS group. TTS: time to surgery.

The median follow-up time was 10.5 months in the standard TTS group and 11.2 months in the prolonged TTS group. A total of five events occurred, with two events in the standard TTS group and three events in the prolonged TTS group, and no significant difference was observed in DFS(P=0.60)(**Figure 4**).

## Discussion

This study indicated no significant differences in pCR rate, DFS, surgical time, lymph nodes dissected number, postoperative hospital stays, total hospital stays, hospital expenses, and CCI, comparing the standard TTS group and the prolonged TTS group after completion of nICT. The postoperative complications and major postoperative complications were comparable in both groups.

The nICT is a novel pattern for locally advanced ESCC. Though treatment-related adverse events (TRAEs) of nICT may occur in a wide range of periods after the application of nICT, especially immune-related pneumonitis (12). Several clinical trials have proved the low severity of treatment-related adverse events (TRAEs) and the manageable of TRAEs. The grade 3 TRAEs were about 10%, and grade 4-5 TRAEs were rare in the nICT pattern (7, 8). In this real-world study, the reasons for prolonged TTS were mostly administrative reasons and the influence of COVID-19 rather than the TRAEs induced by nICT. Due to the limited medical resources, some patients diagnosed with ESCC receiving nICT had to wait more than six weeks. The pandemic of COVI-19 made this contradiction more obvious. Further, based on control policies, patients in medium-high risk areas had to prolong the surgery until the pandemic was controlled. Thus, it’s reasonable to evaluate the impact of prolonged TTS on short-term outcomes.

In this study, pCR rate was the primary outcome, and the pCR rate in both tumor and lymph nodes was 23.08% (12/52) in the standard group and 16.28% (7/43) in the prolonged group (P=0.41). It seems that prolonged TTS couldn’t improve the pCR rate after completion of nICT. To investigate and explain this finding, we refer to the pattern of nCRT. At present, nCRT is still the standard treatment for locally advanced ESCC, and the TTS is recommended 6-8 weeks after completion of nCRT. The pCR is considered a valuable predictor of decreased recurrence and improved long-term survival in ESCC patients undergoing nCRT (13, 14). Shapiro J et al. noted a slightly increased probability of postoperative complications and pCR rate in patients with prolonged TTS after nCRT, without affecting disease-free and overall survival (15). Ranney DN et al. noted that esophageal adenocarcinoma with a longer TTS (≥8 weeks) after nCRT had a higher probability of pathologic downstaging (OR:1.38, 95%CI: 1.02-1.85, P=0.04), and worse long-term survival (hazard ratio 1.44, 95%CI: 1.22-1.71, P<0.001) (16). Qin Q et al. conducted a meta-analysis including 15,086 patients and noted that a TTS longer than 7-8 weeks after was significantly associated with an increased pCR rate (RR, 1.13; 95% CI, 1.05-1.21; P=0.001), comparing with TTS ≤7-8 weeks. Interestingly, Qin Q et al. noted that a prolonged TTS resulted in a poorer 2-year (RR, 0.94; 95% CI, 0.90-0.98; P=0.002) and 5-year OS (RR, 0.88; 95% CI, 0.82-0.95; P=0.0009) (17). Considering the difference between pCR and long-term outcomes in nCRT, the impact of TTS after nICT on recurrence and long-term survival requires further investigation.

Another concern of prolonged TTS is the probability of increased fibrosis. In esophagectomy, operation time is the index of operation difficulty, and the number of lymph node dissections is the index of operation quality. Although previous studies have shown that surgical resection of non-small cell lung cancer (NSCLC) after neoadjuvant immunotherapy is more difficult and challenging due to dense fibrosis, especially mediastinal and hilar dissection (18, 19). A reduction of lymph nodes moved in NSCLC after completion of the neoadjuvant immunotherapy group (20). Based on clinical practice, the fibrosis in esophageal mesangial after nICT was relatively loose, comparing the fibrosis after nCRT (21). In this study, we noted that TTS wasn’t associated with surgical time and lymph nodes dissected number, which indicated that prolonged TTS didn’t increase the probability of dense fibrosis.

The standard group and the prolonged group were similar in postoperative hospital stays, total hospital stays, hospital expenses, and CCI. The details of 30-day complications and major complications were also comparable. The prolonged TTS didn’t increase the risk of postoperative mortality and morbidity. Tie H et al. conducted a meta-analysis including ten retrospective cohort studies and noted that prolonged interval between nCRT and esophagectomy was associated with an increased risk of anastomotic complication (OR 1.71, 95% CI: 1.15 to 2.54, P = 0.008), with no effect on perioperative mortality (OR 1.20, 95% CI 0.79 to 1.83, P = 0.40) (22). Qin Q et al. concluded that prolonged TTS was related to a higher 30-day mortality (RR, 1.51; 95% CI, 1.19-1.92; P = 0.0006) (17). The potential bias of delay-surgery reasons in previous may result in different. Recently, Nilsson K et al. conducted an RCT (including 249 patients with esophageal and esophagogastric junction cancer) to compare the outcomes between standard TTS (4-6 weeks) and prolonged TTS (10-12 weeks) and noted that there were no significant differences between standard TTS and prolonged TTS with regard to overall incidence of complications and major complications, including anastomotic leak (23). To put our results in perspective, we would like to emphasize that most delay-surgery reasons in this study were administrative reasons and the influence of COVID-19 rather than poor physical conditions or TRAEs.

To our best knowledge, this was the study to investigate the impact of TTS after completion of nICT on short-term outcomes. We tried to address potential selection bias through rigorous patient selection based on a prospectively collected database. This study still had the following limitations: Firstly, this study was still couldn’t avoid retrospective nature and only conducted in a single institution, with a relatively limited number of cases. Secondly, due to the limitation of follow-up time and events, we used pCR and DFS to evaluate the anti-tumor effect of nICT, rather than overall survival(OS). Thirdly, although patients included in clinical trials received PET-CT and ultrasound gastroscopy, however, the main staging examination in others was contrast-enhanced CT. Due to the lack of standard staging examinations, the staging would cause some potential bias in conclusion. Fourthly, The administrative reasons and the influence of COVID-19 rather than poor physical conditions or TRAEs were the main reasons for delayed surgery in this cohort. In our opinion, TTS could be extended to 7-9 weeks without significant influence on pathological response and short-term postoperative outcomes. At present, we can’t determine the best time for surgery after neoadjuvant immunotherapy due to the lack of sufficient long-term survival data. To determine the optimal interval, there is an urgent for a larger cohort from multicenter presently, and the next step following well-designed RCTs.

## Conclusions

TTS after completion of nICT is not of major importance with regard to pathological response, disease-free survival, and short-term postoperative outcomes. Considering the distribution of TTS in prolonged groups, we recommend that TTS could be extended to 7-9 weeks when necessary. The impact of TTS on long-term survival should be further investigated.

## Data availability statement

The original contributions presented in the study are included in the article. Further inquiries can be directed to the corresponding authors.

## Author contributions

Z-NH and MK conceived the concept and coordinated the design. Z-NH drafted the manuscript. All authors contributed to the article and approved the submitted version.

## Funding

This study was sponsored by the Startup Fund for Scientific Research at Fujian Medical University (2021QH2022) and Special financial subsidy project of Fujian Province (2020B020).

## Acknowledgments

We deeply appreciate Toedit for its excellent language service.

## Conflict of interest

The authors declare that the research was conducted in the absence of any commercial or financial relationships that could be construed as a potential conflict of interest.

## Publisher’s note

All claims expressed in this article are solely those of the authors and do not necessarily represent those of their affiliated organizations, or those of the publisher, the editors and the reviewers. Any product that may be evaluated in this article, or claim that may be made by its manufacturer, is not guaranteed or endorsed by the publisher.
